# Clinical Outcomes of Thymic Carcinoma: The Role of Radiotherapy Combined with Multimodal Treatments

**DOI:** 10.3390/cancers15082262

**Published:** 2023-04-12

**Authors:** Gowoon Yang, Chang Young Lee, Eun Young Kim, Chang Geol Lee, Min Hee Hong, Byung Jo Park, Hong In Yoon, Kyung Hwan Kim, Sang Hoon Lee, Hwa Kyung Byun, Jaeho Cho

**Affiliations:** 1 Department of Radiation Oncology, Yonsei Cancer Center, Heavy Ion Therapy Research Institute, Yonsei University College of Medicine, 50-1 Yonsei-ro, Seodaemun-gu, Seoul 03722, Republic of Korea; 2 Department of Thoracic and Cardiovascular Surgery, Yonsei University College of Medicine, 50-1 Yonsei-ro, Seodaemun-gu, Seoul 03722, Republic of Korea; 3 Division of Pulmonology, Department of Internal Medicine, Yonsei University College of Medicine, 50-1 Yonsei-ro, Seodaemun-gu, Seoul 03722, Republic of Korea; 4 Division of Medical Oncology, Department of Internal Medicine, Yonsei Cancer Center, Severance Hospital, Yonsei University College of Medicine, 50-1 Yonsei-ro, Seodaemun-gu, Seoul 03722, Republic of Korea

**Keywords:** thymus neoplasms, radiotherapy, combined modality therapy, survival rate

## Abstract

**Simple Summary:**

This study aimed to identify the role of radiotherapy (RT) in the treatment of thymic carcinoma as well as the optimal RT target volume. A total of 116 patients who received multimodal treatment including RT with or without surgery or chemotherapy were included. The RT target volume was defined as the tumor bed or gross tumor with margin, and selective irradiation of the nodal area was performed when involved. The 5-year local recurrence-free survival rate was 94.7%. Overall, 53 recurrences were observed, of which distant metastasis was the most common. No isolated infield or marginal failures were observed. No infield nodal failure occurred among the patients who had initial node metastases and received RT including the nodal areas. A high local control rate was achieved with the addition of RT. A target volume confined to the tumor bed or gross tumor plus margin with the involved lymph node stations seems reasonable.

**Abstract:**

Introduction: We aimed to identify the role of radiotherapy (RT) in the treatment of thymic carcinoma as well as the optimal RT target volume. Materials and Methods: This single-institution retrospective study included 116 patients diagnosed with thymic carcinoma between November 2006 and December 2021 who received multimodal treatment including RT with or without surgery or chemotherapy. Seventy-nine patients (68.1%) were treated with postoperative RT, 17 patients (14.7%) with preoperative RT, 11 patients (9.5%) with definitive RT, and nine patients (7.8%) with palliative RT. The target volume was defined as the tumor bed or gross tumor with margin, and selective irradiation of the regional nodal area was performed when involved. Results: With a median follow-up of 37.0 (range, 6.7–174.3) months, the 5-year overall survival (OS), progression-free survival, and local recurrence-free survival rates were 75.2%, 47.7% and 94.7%, respectively. The 5-year OS was 51.9% in patients with unresectable disease. Overall, 53 recurrences were observed, of which distant metastasis was the most common pattern of failure (*n* = 32, 60.4%) after RT. No isolated infield or marginal failures were observed. Thirty patients (25.8%) who had lymph node metastases at the initial diagnosis had regional nodal areas irradiated. There was no lymph node failure inside the RT field. A tumor dimension of ≥5.7 cm (hazard ratio [HR] 3.01; 95% confidence interval [CI] 1.25–7.26; *p* = 0.030) and postoperative RT (HR 0.20; 95% CI 0.08–0.52; *p* = 0.001) were independently associated with OS. Intensity-modulated-RT-treated patients developed less overall toxicity (*p* < 0.001) and esophagitis (*p* < 0.021) than three-dimensional-conformal-RT-treated patients. Conclusions: A high local control rate was achieved with RT in the primary tumor sites and involved lymph node area in the treatment of thymic carcinoma. A target volume confined to the tumor bed or gross tumor plus margin with the involved lymph node stations seems reasonable. The advanced RT techniques with intensity-modulated RT have led to reduced RT-related toxicity.

## 1. Introduction

Thymic carcinoma is a rare intrathoracic malignancy that usually follows an aggressive clinical course [1]. The treatment strategy for thymic carcinoma is primarily based on whether surgical resection may be achieved. Postoperative radiotherapy (PORT) should be offered to thymic carcinomas at any stage with positive resection margins. Radiotherapy is a useful treatment modality for locally advanced thymic malignancies. Induction chemotherapy or induction chemoradiotherapy followed by surgery may play an important role in the management of locally advanced thymic carcinoma that is considered initially inoperable. Usually, palliative-intent chemotherapy alone is offered to patients with unresectable, metastatic tumors [2]. 

In spite of these current treatment strategies, clinical data remain scarce owing to the lack of prospective clinical trials and possible variation of treatment strategies across institutions [3]. Although an aggressive multimodal treatment, which usually includes surgical resection, radiotherapy (RT), alongside chemotherapy, has been preferred in treating patients with thymic carcinoma, the optimal treatment strategy still needs to be determined. Because of the rarity of the disease, there are currently only small retrospective studies available, and studies with larger sample sizes are needed [4,5,6,7]. 

Few studies have addressed the failure patterns in thymic carcinoma [8]. In this study, we aimed to find out the optimal RT field through a detailed analysis of the pattern of failure. Furthermore, since the role of RT in thymic carcinoma has not yet been well established, this study aimed to find out the role of RT in the treatment of thymic carcinoma through the treatment outcomes of a large number of patients who received RT. 

## 2. Materials and Methods

### 2.1. Patients and Treatment

Patients diagnosed with thymic carcinoma between November 2006 and December 2021 were treated in our institution (*n* = 162). Patients who did not receive RT or who received RT only for lesions other than the thymic lesions were excluded (*n* = 46). Thus, we analyzed 116 patients with thymic carcinoma treated with postoperative, preoperative, definitive, or palliative RT with or without a combination of surgery and/or chemotherapy. This study was approved by the Institutional Review Board of Severance Hospital, which waived the requirement for informed patient consent (4-2022-0961). The patient and treatment characteristics are summarized in Table 1. The median age was 59 years (range, 22–83 years), and 67.2% of the patients were men. Eighty-two patients (70.7%) presented with European Cooperative Oncology Group (ECOG) stage 1 and 29 (25.0%) with ECOG stage 0. The Masaoka–Koga stage was II in 49 patients (42.2%), III in 23 (19.8%), IVA in 14 (12.1%), and IVB in 30 (25.9%). The median tumor size was 5.7 cm (range, 1.8–16.6 cm).

### 2.2. Treatment Characteristics

#### 2.2.1. Surgery

Surgery, radiation, and chemotherapy treatment details according to the Masaoka stage are shown in Table 2. Surgery was performed in 93 patients (80.2%). The postsurgical residual tumor burden was R0 in 65 patients (56.0%), R1 in 19 (16.4%), and R2 in 7 (6.0%).

#### 2.2.2. Radiotherapy

Seventy-nine patients received postoperative aim RT (68.1%), 17 received preoperative aim (14.7%), 11 received definitive aim (9.5%), and 9 received palliative aim (7.8%). IMRT was used in 83 patients (71.6%), and the remaining patients (27.6%) received 3D-CRT. 

Definitive RT was defined as when RT was performed including all the existing lesions, without undergoing surgical resection. Palliative RT was defined as when RT was performed not including all the existing lesions, without undergoing surgical resection and the lesions to be treated were determined at the discretion of the radiation oncologist. Among patients with Masaoka stage II, 46 patients (93.8%) received postoperative RT and 3 patients (6.1%) received preoperative RT. Among patients with Masaoka stage III, 17 patients (73.9%) received postoperative RT and 5 patients (21.7%) preoperative RT. In patients diagnosed with Masaoka stage IVA, six patients (42.9%) were treated with postoperative RT, four patients (28.6%) with definitive RT, and three patients (21.4%) with palliative RT. In patients diagnosed with Masaoka stage IVB, 10 patients (33.3%) were treated with postoperative RT, 8 patients (26.7%) with preoperative RT, 6 patients (20.0%) with definitive RT, and 6 patients (20.0%) with palliative RT. 

The gross target volume (GTV) was defined as the gross tumor, in cases of preoperative or definitive RT, or the tumor bed including the initially involved pleura observed on preoperative computed tomography (CT) and positron emission tomography (PET-CT) performed prior to surgery in cases of postoperative RT. The clinical target volume (CTV) was created by the symmetrical expansion of the tumor bed by 1 cm in all directions, with a margin of 2 cm alongside the adjacent pleura. The internal target volume (ITV) was created including CTV with a margin that reflected the movement of the heart and lungs. The planning target volume (PTV) was created by the symmetrical expansion of CTV by 3–5 mm in all directions. The regional nodal areas were covered when involved at initial diagnosis. The lymph node stations were delineated in accordance with a contouring atlas [9,10,11]. For patients treated with palliative RT, the target volume included the primary tumor and the PTVs were created in the same manner as described above. Eighty-three patients (71.6%) were treated with IMRT and 32 patients (27.6%) were treated with 3D-CRT using three anteriorly located beams (anterior and bilateral anterior oblique). The dose and fractionation varied according to the treatment aim. For postoperative aim, the median total dose was 50.2 Gy (45–63 Gy), delivered at 1.8–2 Gy per fraction. For definitive aim, the median dose was 63 Gy (50–68.6 Gy), delivered at 1.8–2 Gy per fraction.

#### 2.2.3. Chemotherapy

Fifty-seven patients received chemotherapy (49.1%). The most commonly used chemotherapy combinations were etoposide and cisplatin (36.2%), followed by cisplatin, adriamycin (doxorubicin), and cyclophosphamide (6.9%). 

### 2.3. Endpoints and Statistical Analyses

The failure patterns were classified according to the International Thymic Malignancy Interest Group (ITMIG) guidelines [12]: local, regional, and distant failure. Local failure was defined as failure within or around the primary tumor bed. Regional failure was defined as failure that occurred within the thorax, not contiguous with the primary tumor or tumor bed including pleuropericardial seeding. Distant failure was defined as failure that occurred outside the thorax including lung metastasis. Additionally, all treatment failures were subdivided according to the ITMIG guidelines [13]: (1) infield failures (within the 100% isodose line [IDL]), (2) marginal recurrences (<100% and ≥50% IDL), and (3) outfield failures (outside the 50% IDL). The OS duration was calculated from the initial histopathologic diagnosis until the last follow-up or death, and the PFS duration was defined from the time of the RT finish to the time of disease progression or death. LRFS was calculated from the time of the RT finish until the date of local failure. The Kaplan–Meier analysis was performed to determine survival rates. The prognostic factors for 5-year OS and PFS were analyzed using univariate and multivariate Cox regression analyses. Adverse events were graded according to the Common Terminology Criteria for Adverse Events (CTCAE), version 4.0. The chi-square test and t-test were performed to analyze the distributions of categorical and continuous variables, respectively. *p*-values of <0.05 were considered statistically significant, and SPSS (version 23.0; IBM Corp., Armonk, NY, USA) was used for the statistical analyses.

## 3. Results

### 3.1. Survival Outcomes

**Figure 1:**
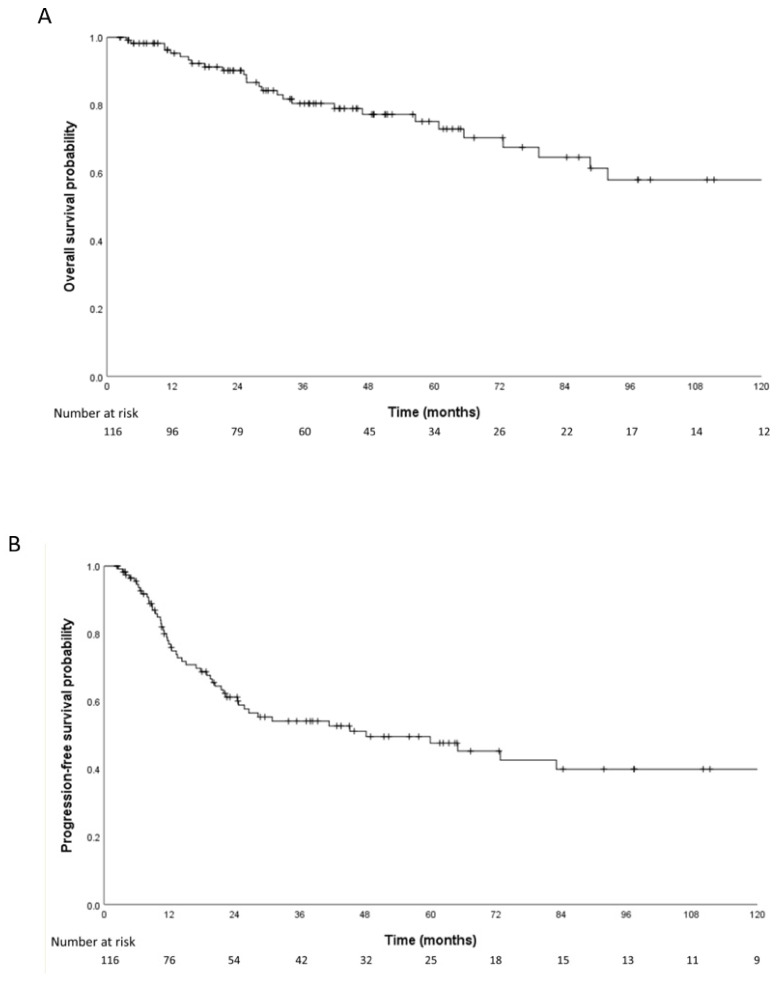

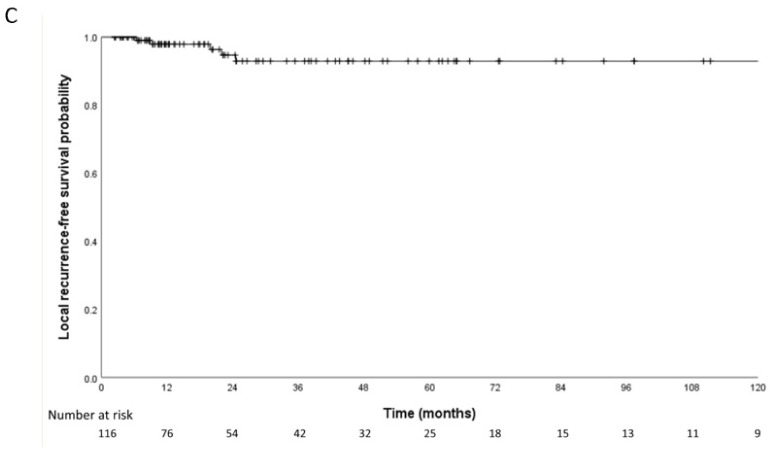
Kaplan–Meier survival curves for overall survival (**A**), progression-free survival (**B**), and local recurrence-free survival (**C**).

The median follow-up period was 37.0 months (range, 6.7–174.3 months), and 29 deaths and 53 recurrences were observed. The median OS and PFS durations were 163.1 months (95% confidence interval [CI], 62.5–263.7) and 48.1 months (95% CI, 11.7–84.6). The median LRFS duration was not determined. The 5-year OS, PFS, and LRFS rates were 75.2%, 47.7% and 94.7%, respectively (Figure 1).

### 3.2. Patterns of Failure in Nodal Metastasis

**Figure 2:**
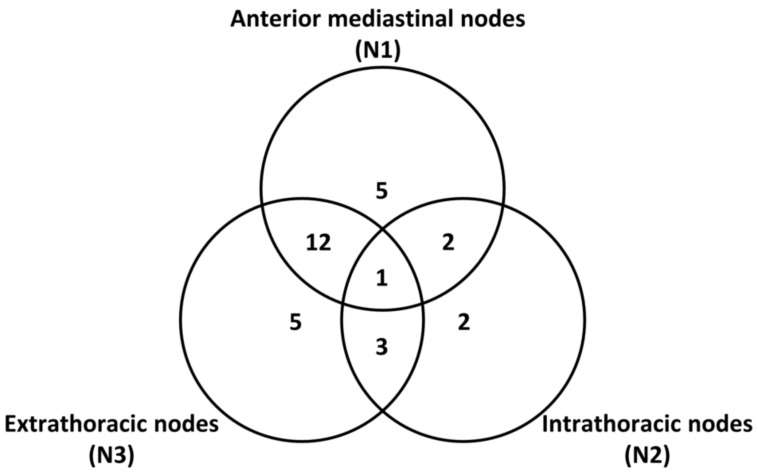
Patterns of nodal involvement at initial diagnosis (*n* = 30). Anterior mediastinal nodes (N1), intrathoracic nodes (N2), and extrathoracic nodes (N3).

Regional lymph nodes of the thymus were classified into three groups: anterior mediastinal (N1), intrathoracic excluding the anterior mediastinal (N2), and extrathoracic (N3), as described previously [14]. Thirty patients (25.8%) had lymph node metastases at the initial diagnosis. Nodal metastasis consisted of 20 N1 (66.7%), 7 N2 (23.3%), and 21 N3 (70.0%) metastases. In patients with N1 metastasis, 5 patients had N1 metastasis alone, 3 patients had both N1 and N2 metastasis, 13 patients had both N1 and N3 metastasis, and 1 patient had N1, N2, and N3 metastasis. In patients with N2 metastasis, two patients had N2 alone and three patients had both N2 and N3 metastasis. Five patients with N3 metastasis had N3 metastasis alone (Figure 2). The RT field covered regional nodal areas in these patients. There was no lymph node failure inside the RT field. After RT, 6 patients (20.0%) experienced outfield nodal metastasis, among the 30 patients who had initial nodal metastases. The details of each patient is described in Supplemental Table S1. In the 17 patients (14.7%) who were irradiated including the involved supraclavicular area, nodal metastasis occurred in 1 patient (pericardial lymph node [N2]) and no failure occurred within the ipsilateral supraclavicular fossa. The median dose applied to the supraclavicular fossa was 55 Gy (50–68.6 Gy), delivered in 25–32 fractions.

### 3.3. Patterns of Failure

Table 3 provides detailed information on the failure patterns. No isolated infield or marginal failure was observed in the RT field. Outfield failure was the most common pattern of failure (*n* = 47, 88.7%), followed by marginal+outfield failure (*n* = 5, 9.4%) and infield+outfield failure (*n* = 1, 1.9%). Distant failure was the most common (*n* = 32, 60.4%), followed by regional failure (*n* = 11, 20.8%) and regional+distant failure (*n* = 9, 17.0%). Among the patients who experienced exclusively outfield failure, the most common site was the lung parenchyma (*n* = 31, 58.5%), followed by the pleura (*n* = 20, 37.7%). In patients who were treated with definitive or palliative RT without surgery (*n* = 23), disease progression was observed in 18 patients (78.3%) with no local, infield, or isolated regional failure. Among these patients, 15 (83.3%) experienced isolated distant failure and 5 (16.7%) experienced regional and distant failure.

### 3.4. Prognostic Factors

We analyzed the significance of potential prognostic factors regarding the 5-year OS and PFS rates (Table 4). In multivariate analysis, a tumor dimension of ≥5.7 cm (hazard ratio [HR] 3.01; 95% confidence interval [CI] 1.25–7.26; *p* = 0.030) was associated with poor OS and postoperative RT was associated with favorable OS (HR 0.20; 95% CI 0.08–0.52; *p* = 0.001). Non-R0 resection (HR 2.55; 95% CI 1.38–4.71; *p* = 0.003) was associated with poor PFS and postoperative RT was associated with favorable PFS (HR 0.34; 95% CI 0.18–0.64; *p* = 0.001). 

### 3.5. Toxicity

Regarding acute toxicity, no patient developed grade 3 or higher RT-related toxicity according to CTCAE version 4.0 (Supplemental Table S2). Overall, 18 patients (21.7%) treated with IMRT and 23 (69.7%) treated with 3D-CRT developed grade 1 or 2 RT-related toxicities (*p* < 0.001). Among them, eight patients (9.8%) treated with IMRT and five (15.2%) treated with 3D-CRT developed grade 1 or 2 radiation pneumonitis (*p* = 0.157). Four IMRT-treated (4.8%) and six 3D-CRT-treated patients (18.2%) developed grade 1 or 2 esophagitis (*p* = 0.021).

## 4. Discussion

Thymic carcinoma is an aggressive malignancy usually leading to poorer clinical outcomes than types A to B3 thymic epithelial tumors. Several recent population-based studies have shown that RT in patients who underwent surgery improves OS and/or recurrence-free survival [15,16,17,18]. The 5-year OS rate in our study was 75.2%, which was favorable compared with that in population-based studies (61–63%) [3,12] and other retrospective studies (53–68%) [19,20]. The failure patterns showed that RT is an effective treatment modality for local control. 

Lee et al. [8] reported failure patterns after PORT in relation to the target volume in thymic carcinoma and concluded that the policy of PORT target volume confined to only the tumor bed seems reasonable. However, the failure pattern according to the target volume after RT delivered in various clinical settings has not been studied previously. Consistent with the findings of Lee et al. [8], the main failure pattern after RT in the present study was outfield failure, observed in 47/53 (88.7%) patients who experienced any type of failure. No isolated infield or marginal failures were observed in our study. No local, infield, or isolated regional failures were observed in the subgroup of patients who were treated with definitive or palliative RT without surgery. This implies that a high local control rate was achieved with RT, despite the high proportion of patients who were not surgical candidates. The current policy of a target volume confined to the tumor bed or gross tumor plus margin seems reasonable. 

Patterns of lymphogenous metastasis were also analyzed. The nodal metastasis rate (25.8%) was similar to previously reported rates (25.0–26.8%) [14,21]. Notably, the rate of extrathoracic node metastasis at the initial diagnosis (70.0%) was higher than previously reported rates (30.6%) [14], suggesting that a significant number of advanced-stage disease patients was included in our study. Regional nodal areas were selectively covered only when involved at the initial diagnosis. Patterns of nodal metastasis after RT in patients who were node positive status at the initial diagnosis were also analyzed. In all patients who experienced subsequent nodal metastasis after RT, recurrence occurred at stations other than the initial nodal station. All patients with supraclavicular lymph node metastasis at the initial diagnosis received RT including the involved supraclavicular area. Among these patients, 35.3% of the patients experienced progression: mostly exclusively distant metastasis (66.7%). No failures occurred within the ipsilateral supraclavicular fossa. These findings imply that a high local control rate can be achieved with RT including the involved lymph node stations. Therefore, the current policy of a target volume confined to the involved lymph node stations seems reasonable. 

In the entire cohort, recurrence was observed in 41 patients (68.3%), and distant failure was the most common failure pattern (*n* = 29, 70.7%). Therefore, chemotherapy did not effectively control the disease outside the RT field. The most commonly used combination of chemotherapy was etoposide and cisplatin (36.2%) followed by cisplatin, adriamycin (doxorubicin), and cyclophosphamide (9.5%). The role of molecular-targeted therapy to treat unresectable TC was evaluated in a phase 2 clinical trial [22]. Recent studies on immunotherapeutic target molecules in thymic carcinoma have shown frequent expression of programmed death 1 (PD-1) and programmed death ligand 1 (PD-L1) in thymic carcinoma and an association of PD-L1 expression with improved survival. Therefore, anti PD-1/PD-L1 therapies should be developed [20,23]. There are several phase II studies of PD-1 blockade monotherapy in patients with thymic carcinoma currently ongoing [24]. In addition, the effect of a combination therapy of tyrosine kinase inhibitors with PD-1 blockade is also being examined as a targeted treatment for thymic carcinoma (NCT03463460). Continuous efforts to develop more effective systemic therapies that can improve survival are essential. 

A smaller greatest tumor dimension and upfront surgery were independently associated with improved OS. Surgical status and upfront surgery were independently associated with PFS. Complete surgical resection has been reported to be an important prognostic factor [25]. The rate of complete resection varies from 20% to 88% in published series [26,27,28,29,30,31]. In the present study, 56.0% of cases underwent an R0 resection and the rate of complete resection decreased with increasing stage. Patients who underwent an R0 resection had a higher 5-year (68.8% vs. 33.9%, *p* = 0.021) PFS compared to patients with an R1/2 resection. Therefore, complete resection seems to lead to long-term recurrence-free survival even in locally advanced thymic carcinomas. Thus, the goal of any surgical approach should be an R0 resection. In multivariate analysis, chemotherapy was not associated with OS or PFS, which differs from previous reports where chemotherapy seemed to have benefit in both surgical and nonsurgical settings [32,33]. 

In this study, patients were treated with IMRT (71.6%) or 3D-CRT (27.6%). IMRT-treated patients developed less overall toxicity (*p* < 0.001) and esophagitis (*p* < 0.021) than 3D-CRT-treated patients. The increased esophageal toxicity was because the target volume occasionally included a considerable portion of the anterior mediastinum. It was demonstrated that esophageal sparing was possible with IMRT. In a recent phase 3 randomized clinical trial which compared esophageal-sparing IMRT and 3D-CRT in patients with stage III/IV incurable non–small cell lung cancer, esophageal-sparing IMRT significantly reduced symptomatic esophagitis (24% [*n* = 11] vs. 2% [*n* = 1], *p* = 0.002) [34]. Moreover, the mean lung dose was lower in patients treated with IMRT than in those treated with 3D-CRT, although the difference was not significant (8.38 vs. 10.31 Gy, *p* = 0.090). Sparing the lung parenchyma was technically possible with IMRT, especially when the shape of the target was concave (Supplemental Figure S1a,b). The advancement of high-precision RT techniques with IMRT has led to reduced treatment-related toxicity. 

The survival rates were favorable compared with those in previously reported studies [16,18,19,35]. A relatively higher proportion of patients who underwent R0 to R1 resection may be one of the causes of improved OS. Importantly, we obtained a comparable result despite the higher proportion of patients with Masaoka–Koga stage IVB (25.9%), who are usually not eligible for surgery because of the advanced-stage disease. Compared to a recent study which reported survival outcomes following postoperative RT in thymic carcinoma and thymic neuroendocrine tumors, although the proportion of patients with Masaoka–Koga stage IVB was much higher in our study (25.9% vs. 3.8%), the 5-year rates of OS and PFS were comparable (75.2% vs. 81.0% and 47.7% vs. 49.7%, respectively) [8]. This reflects that the use of RT can be of benefit even in patients with Masaoka stage IV thymic carcinoma. In addition, Lim et al. [18] reported that OS improved with postoperative RT compared with surgery alone (63.2% vs. 50.5%, *p* = 0.007). Moreover, adjuvant therapy improved survival compared with surgery alone in patients with Masaoka–Koga stages IIB (median OS 106.0 months vs. not reached, *p* = 0.010) and III (median OS 64.8 months vs. 94.9 months, *p* < 0.010) in Kim et al. [36]’s report. Furthermore, a 5-year OS of 27.0% was reported in patients with unresectable disease when RT was performed in only 49.0% of patients in Weksler et al. [27]’s report. In our study, the 5-year OS reached 51.9% even in patients without surgery. Therefore, incorporating RT into the multidisciplinary management of thymic carcinoma can improve patient survival. In particular, our results suggest that even in patients with advanced-stage disease, treatment results can be improved by active implementation of RT instead of relying only on chemotherapy. 

The major limitations of this analysis are its retrospective nature. This was a retrospective, non-randomized study subject to selection bias or the influence of unknown variables. Nevertheless, our study represents one of the largest series of thymic carcinoma to date. Considering that most of the existing literature related to thymic carcinoma are small retrospective studies [4,5,6,7], the results of this study holds clinical significance. In addition, the patterns of node metastasis in thymic carcinoma are not well known, unlike thymoma, and the patterns of node metastasis at the initial diagnosis and after treatment were analyzed in this study. 

## 5. Conclusions

The analysis of patterns of failure in regard to the target volumes and patterns of node metastasis revealed that the current policy of a target volume confined to GTV plus margin with the involved lymph node stations is reasonable. A high local control rate was achieved with the addition of RT even in stage IVB patients. This finding holds great clinical significance as an excellent OS was obtained with a multimodal treatment including RT. Our results suggest that treatment outcomes can be improved by active implementation of RT even in patients with advanced-stage disease. 

## Figures and Tables

**Table 1:** Patient and treatment characteristics.

Variables	*n*	%
Age, median (IQR), y	59 (48.3–67.0)	
Sex		
Male	78	67.2%
Female	38	32.8%
ECOG		
0	29	25.0%
1	82	70.7%
2	5	4.3%
Masaoka–Koga stage		
II	49	42.2%
III	23	19.8%
IVA	14	12.1%
IVB	30	25.9%
Greatest tumor dimension, median (IQR), cm	5.7 (4.0–7.6)	
Surgical status		
No surgery	23	19.8%
R0	65	56.0%
R1	19	16.4%
R2	7	6.0%
Surgery but unknown	2	1.7%
RT aim		
Postoperative	79	68.1%
Preoperative	17	14.7%
Definitive	11	9.5%
Palliative	9	7.8%
RT modality		
3D-CRT	32	27.6%
IMRT	83	71.6%
Unknown	1	0.9%
Chemotherapy		
No	59	50.9%
Yes	57	49.1%
Chemotherapy regimen		
No chemotherapy	59	50.9%
Cisplatin/Adriamycin/Cyclophosphamide	8	6.9%
Etoposide/Cisplatin	42	36.2%
Others	7	6.0%

ECOG, European Cooperative Oncology Group; RT, radiotherapy; 3D-CRT, three-dimensional conformal radiotherapy; IMRT, intensity-modulated radiotherapy.

**Table 2:** Use of treatment modalities according to the Masaoka stage.

		Stages	Stage II	Stage III	Stage IVA	Stage IVB
Treatment Modalities		*n* (%)	49 (42.2)	23 (19.8)	14 (12.1)	30 (25.9)
Surgery + Post-op RT			46 (93.8)	17 (73.9)	6 (42.9)	10 (33.3)
Chemotherapy	Yes		3 (6.1)	8 (34.7)	4 (28.6)	7 (23.3)
	No		43 (87.8)	9 (39.1)	2 (14.3)	3 (10.0)
Pre-op RT + Surgery			3 (6.1)	5 (21.7)	1 (7.1)	8 (26.7)
Chemotherapy	Yes		3 (6.1)	5 (21.7)	1 (7.1)	8 (26.7)
	No		0 (0.0)	0 (0.0)	0 (0.0)	0 (0.0)
Definitive RT			0 (0.0)	1 (4.3)	4 (28.6)	6 (20.0)
Chemotherapy	Yes		0 (0.0)	1 (4.3)	4 (28.6)	6 (20.0)
	No		0 (0.0)	0 (0.0)	0 (0.0)	0 (0.0)
Palliative RT			0 (0.0)	0 (0.0)	3 (21.4)	6 (20.0)
Chemotherapy	Yes		0 (0.0)	0 (0.0)	3 (21.4)	6 (20.0)
	No		0 (0.0)	0 (0.0)	0 (0.0)	0 (0.0)

RT, radiotherapy.

**Table 3:** Failure patterns according to ITMIG guidelines.

	Local	Local +Regional	Regional	Regional +Distant	Distant	Total
Failure pattern, n						
Infield	0	0	0	0	0	0
Infield + outfield	0	1	0	0	0	1
Marginal	0	0	0	0	0	0
Marginal + outfield	0	0	0	5	0	5
Outfield	0	0	11	4	32	47
Total, n	0	1	11	9	32	53

ITMIG, International Thymic Malignancy Group.

**Table 4:** Multivariate analysis for overall survival and progression-free survival.

Variables	OS		PFS	
	HR (95% CI)	*P*	HR (95% CI)	*p*
Age (per 1y)	1.00 (0.97–1.03)	0.733	1.00 (0.97–1.01)	0.308
Female sex (vs. male)	0.83 (0.37–1.87)	0.648	1.28 (0.73–2.23)	0.384
ECOG ≥ 2 (vs. 0–1)	2.20 (0.68–12.53)	0.150	1.63 (0.51–5.24)	0.414
Masaoka–Koga stage III, IV (vs. IIA, IIB)	1.41 (0.56–3.59)	0.465	0.99 (0.48–2.06)	0.975
Greatest tumor dimension ≥5.7 cm (<5.7 cm)	3.01 (1.25–7.26)	0.014	1.37 (0.77–2.45)	0.289
Initial surgery yes (vs. no)	0.62 (0.16–2.41)	0.493	0.81 (0.32–2.05)	0.662
Surgical status R1, R2, No surgery (vs. R0)	0.95 (0.34–2.61)	0.937	2.55 (1.38–4.71)	0.003
Chemotherapy yes (vs. no)	2.41 (0.76–7.64)	0.135	1.68 (0.72–3.90)	0.226
Radiotherapy aim Postoperative (vs. others)	0.20 (0.08–0.52)	0.001	0.34 (0.18–0.64)	0.001
IMRT (vs. 3D-CRT)	1.45 (0.62–3.38)	0.394	0.76 (0.32–1.78)	0.527

OS, overall survival; PFS, progression-free survival; HR, hazard ratio; CI, confidence interval; ECOG, European Cooperative Oncology Group; IMRT, intensity-modulated radiotherapy; 3D-CRT, three-dimensional conformal radiotherapy.

## Data Availability

Not applicable.

## Supplementary Materials

The following supporting information can be downloaded at: https://www.mdpi.com/article/10.3390/cancers15082262/s1, Figure S1a: A representative case of intensity-modulated radiotherapy plan using simultaneous integrated boost technique; Figure S1b: A representative case of three-dimensional conformal radiotherapy plan; Table S1: Details of failed regional lymph nodes after radiotherapy in patients with regional lymph node metastasis at initial diagnosis; Table S2: Toxicity according to treatment modalities.

## Abbreviations

3D-CRT	three-dimensional conformal radiotherapy
ECOG	European Cooperative Oncology Group
IMRT	intensity-modulated radiotherapy
ITMIG	International Thymic Malignancy Interest Group
ITV	internal target volume
LRFS	local recurrence-free survival
OS	overall survival
PD-1	programmed death 1
PD-L1	programmed death ligand 1
PFS	progression-free survival
PTV	planning target volume
RT	radiotherapy
