# Supplementary material for: Clinical Outcomes of Thymic Carcinoma: The Role of Radiotherapy Combined with Multimodal Treatments
Source: Cancers (Basel). 2023 Apr 12;15(8):2262. doi: 10.3390/cancers15082262 (PMC10136483; doi:10.3390/cancers15082262)
Supplement: Supplementary file 1 [file cancers-15-02262-s001.zip › cancers-2300088-supplementary.pdf]

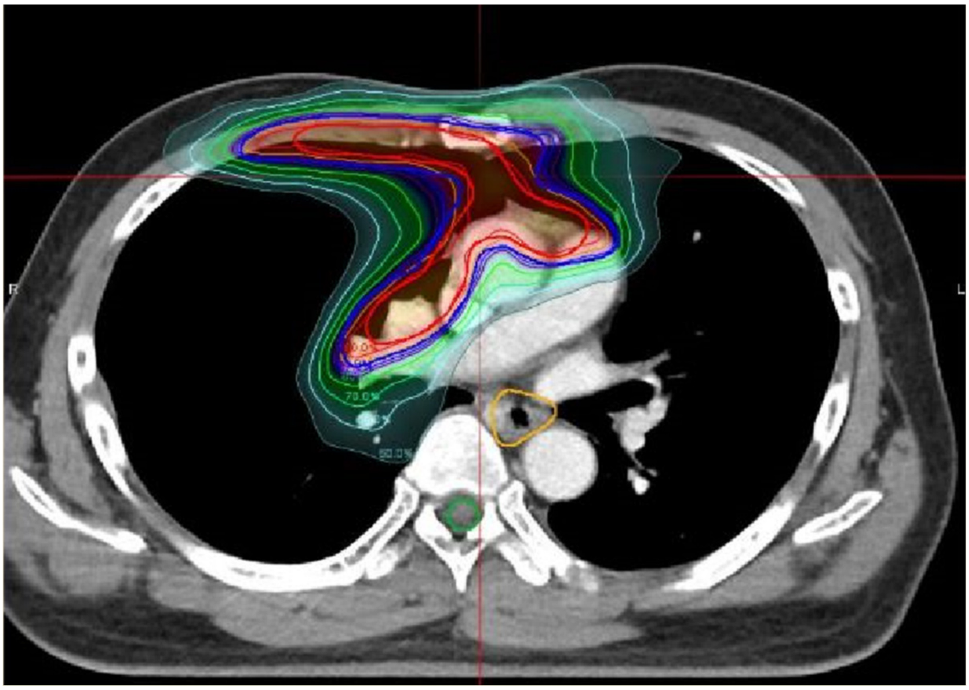

Figure S1. a. A representative case of intensity-modulated radiotherapy plan using simultaneous integrated boost technique.

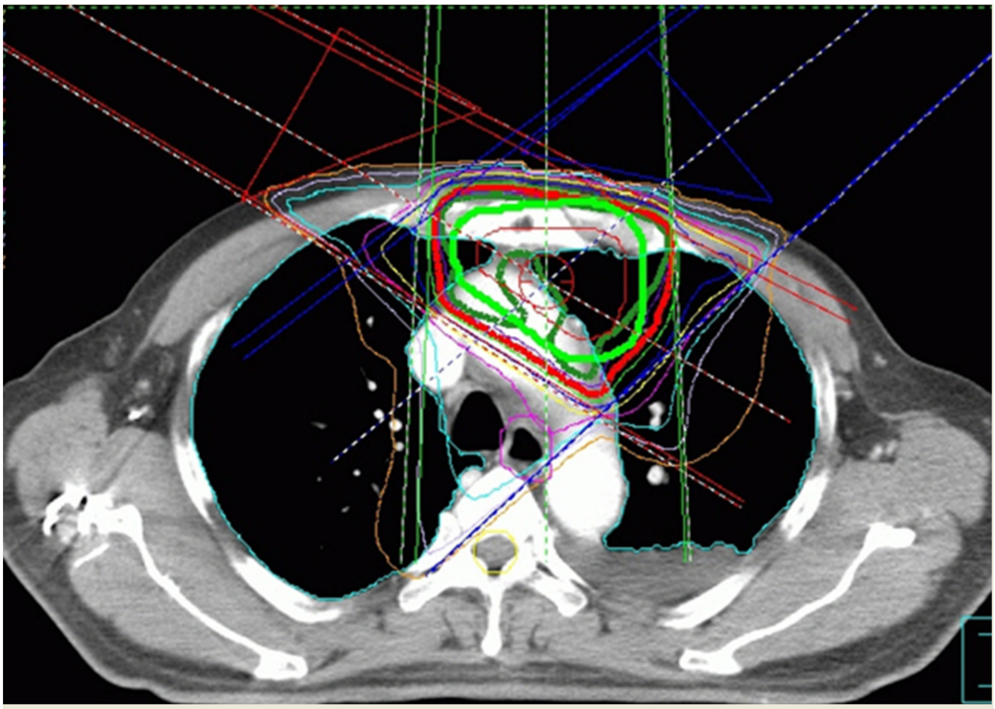

Figure S1. b. A representative case of three-dimensional conformal radiotherapy plan.

Table S1. Details of failed regional lymph nodes after radiotherapy in patients with regional lymph node metastasis at initial diagnosis.

	**Involved regional lymph nodes at initial diagnosis**	**Failed regional lymph nodes after RT**	**RT dose (Gy)**
Patient 1	N2 (Hilar, Lt. Interlobar lymph node)	N2 (Outfield, Lt. Paratracheal lymph node)	50
Patient 2	N3 (Rt. Neck level V lymph node)	N3 (Outfield, Lt. Neck node)	54
Patient 3	N1, N3 (Lt. Neck, cardiophrenic angle, Lt. Supraclavicular lymph node)	N2 (Outfield, Pericardial)	59.4
Patient 4	N3 (Rt. Neck node)	N2, N3 (Outfield, Pericardial, Lt. Neck node)	64
Patient 5	N2 (Mediastinal, paratracheal, retrosternal lymph node)	N2 (Outfield, Subcarinal lymph node)	60
Patient 6	N1, N2 (Internal mammary, both paratracheal, prevascular lymph node)	N3 (Outfield, Rt. Neck node)	50

RT, radiotherapy; Lt, Left; Rt, Right.

Table S2. Toxicity according to treatment modalities.

**Variables, n (%)**	**IMRT (n=83)**	**3D-CRT (n=33)**	***P***
Overall toxicity	18 (21.7)	23 (69.7)	<0.001
No toxicity	65 (78.3)	10 (30.3)	
Radiation pneumonitis	8 (10.7)	5 (15.2)	0.157
No radiation pneumonitis	75 (90.4)	28 (84.8)	
Esophagitis	4 (4.8)	6 (18.2)	0.021
No esophagitis	79 (95.2)	27 (81.8)	

IMRT, intensity-modulated radiotherapy; 3D-CRT, three-dimensional conformal radiotherapy.
